# Network-Guided Identification of Plant-Derived Modulators of Stress-Adaptive Signalling in Neuroblastoma

**DOI:** 10.3390/ijms27093739

**Published:** 2026-04-23

**Authors:** Mmei Cheryl Motshudi, Clarissa Marcelle Naidoo, Chikwelu Lawrence Obi, Benson Chucks Iweriebor, Earl Prinsloo, Muhammad Sulaiman Zubair, Nqobile Monate Mkolo

**Affiliations:** 1 Department of Biology, School of Science and Technology, Sefako Makgatho Health Science University, Pretoria 0204, South Africa; cheryl.motshudi@smu.ac.za (M.C.M.); clarissa.naidoo@smu.ac.za (C.M.N.); lawrence.obi@smu.ac.za (C.L.O.); benson.iweriebor@smu.ac.za (B.C.I.); 2 Department of Biotechnology, Rhodes University, Makhanda 6140, South Africa; e.prinsloo@ru.ac.za; 3 Department of Pharmacy, University of Tadulako, Palu 94118, Indonesia; sulaimanzubair@untad.ac.id

**Keywords:** neuroblastoma, doxorubicin, stress adaptation, plant metabolites

## Abstract

Neuroblastoma is characterized by noticeable resistance to chemotherapy, largely driven by the ability of tumour cells to reorganize stress-adaptive signalling networks rather than relying on single oncogenic drivers. We conducted a study to investigate the pharmacological mode of action of doxorubicin in modifying adaptive signalling pathways in SH-SY5Y neuroblastoma cells, and whether the capacity of plant metabolites can exploit emergent biochemical vulnerabilities. Transcriptomic profiling through RNA sequencing conducted 48 h post-doxorubicin exposure unveiled the organized disruption of pathways linked with amyloidogenic processes, oncogenic signalling pathways, oxidative stress, and DNA repair. The protein–protein interactions, coupled with Kyoto Encyclopedia of Genes and Genomes pathway evaluations, revealed five network-central-hubs: BRAF, GSK3β, PARP1, BACE1, and MAOB. Structural docking integrated with 200 ns molecular dynamics simulations illustrated binding stability across multiple targets driven by three metabolites, Lactol binding to BRAF (−54.13 kcal/mol) and MAOB (−39.08 kcal/mol), Amino(1H-indol-2-yl)acetic acid to BACE1 (−41.07 kcal/mol) and GSK3β (−47.38 kcal/mol), and Quercetin-3-(6″-malonyl-glucoside) binding to PARP1 (−46.03 kcal/mol). In vitro Cell Counting Kit-8 proliferation assays validated the significant anti-neuroblastoma efficacy, with the lowest IC_50_ (0.2397 µM) being exhibited by Amino(1H-indol-2-yl)acetic acid, followed by Lactol (1.226 µM) and Quercetin-3-(6″-malonyl-glucoside) (1.301 µM), which mirrored the cytotoxic action of doxorubicin (1.306 µM). These results suggest that plant-derived metabolites may interact with stress-adaptive signalling pathways connected with neuroblastoma. However, direct experimental validation of target engagement and pathway modulation will be required to confirm these predicted interactions.

## 1. Introduction

Neuroblastoma is a pediatric solid tumour that continues to rank as fatal, not as a consequence of the absence of treatment targets, but due to cancer cells stemming from a neural lineage that depicts an extraordinary ability to endure integrated cytotoxic stress responses through coordinated adaptations of the molecular structure [1,2,3]. Neuroblastoma accounts for approximately 7–10% of paediatric malignancies but it is responsible to nearly 15% of childhood cancer-related mortality worldwide [4,5]. Prognosis varies markedly by risk category, and survival exceeds 90% in low-risk disease but remains below 50% in high-risk neuroblastoma in spite of multimodal therapy [5,6]. Resistance to chemotherapy continues to represent a major clinical challenge and is progressively associated with adaptive stress-response signalling networks that permit tumour cells to tolerate genotoxic and oxidative stress induced by treatment [7,8,9,10]. These adaptive responses encompass coordinated regulation of DNA-damage repair pathways [9], mitochondrial metabolic reprogramming [7], pro-survival kinase signalling [8], and maintenance of redox homeostasis [10], thereby promoting tumour persistence and relapse.

Despite extensive oxidative stress and significant DNA damage induced by anthracycline-based chemotherapeutics, numerous tumours evade destruction by restructuring their transcriptional and signalling networks into robust survival configuration states that promote persistence and recurrence [1,10,11]. Among the treatment of neuroblastoma, anthracycline agents such as doxorubicin are well-characterized inducers of DNA damage and oxidative stress, leading to adaptive transcriptional and metabolic reprogramming. By triggering DNA damage response pathways, redox regulatory mechanisms, and pro-survival kinase signalling cascades, doxorubicin offers a relevant model for investigating therapy-induced stress adaptation at the systems level [1,9,12,13].

An increasing body of evidence suggests that resistance is not solely instigated by isolated oncogenic drivers but by complex, interrelated stress-response networks that comprise mitochondrial metabolism, kinase signalling, and DNA repair [7,10,11,14]. Consequently, comprehending and exploiting this network-level plasticity, therapeutically, emerges as a pivotal challenge in the management of high-risk neuroblastoma [1,3].

Stress from chemotherapy treatment activates a wide spectrum of protective pathways that allow tumour cells to mitigate oxidative and genotoxic damages encompassing improved DNA damage response signalling, mitochondrial maintenance, and reconfiguration of redox balance [7,11]. These adaptive mechanisms in neuroblastoma are influenced by the neural lineage of the tumour, wherein developmental and neuroprotective pathways intersect with oncogenic signalling, thereby establishing a favourable environment for therapeutic resistance [3,14]. As a result, the tumour cells that survive often maintain a hybrid stress phenotype that facilitates sustained proliferation as well as resistance to apoptosis during prolonged drug exposure [1,15].

These findings bolster a model of systemic network addiction wherein cancer cells depend on the synchronized function and activity of numerous stress-adaptive pathways instead of a singular dominant driver [7,15]. Such interdependencies suggest that multi-target modulation might represent a promising therapeutic strategy. Natural products and plant-derived metabolites offer chemically diverse molecular scaffolds that may provide candidates for multi-target engagement; yet bioactivity cannot be inferred and requires systematic identification, prioritization, and experimental validation [1,10]. In the current study, the emphasis on plant-derived metabolites reflects a metabolomics-guided screening strategy as opposed to an assumption of exclusive bioactivity, with compound prioritization informed by differential abundance, pathway relevance, and network topology. Moreover, transcriptomic profiling, protein–protein interaction analysis, functional pathway enrichment, and in vitro pharmacology were integrated to delineate the stress-adaptive landscape triggered by doxorubicin in SH-SY5Y neuroblastoma cells and to uncover plant-derived metabolites with anticipated interaction within essential network hubs. To model this adaptive response in vitro, SH-SY5Y human neuroblastoma cells, derived from a bone marrow metastasis of a neuroblastoma patient, were selected as an experimental model, since they are utilized for investigating oxidative stress, mitochondrial dysfunction, and stress signalling in neuronal and oncological research, including studies of cellular responses to genotoxic insults and adaptive survival mechanisms [16,17].

Together, this integrative framework seeks to identify stress-adaptive signalling pathways in neuroblastoma and explore possible interactions between plant-derived metabolites and key network components, thereby connecting network biology with molecular pharmacology.

## 2. Results

### 2.1. RNA Quality Assessment

**Figure 1:**
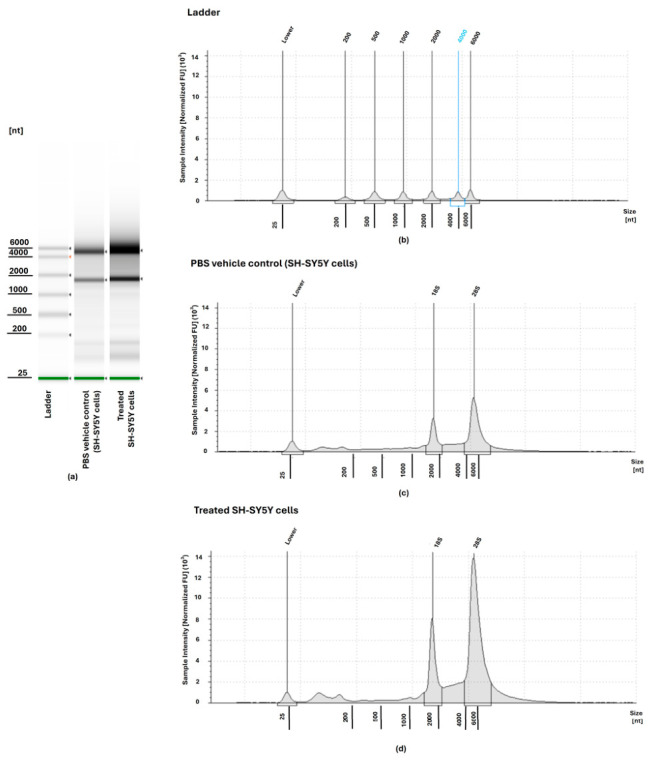
RNA integrity assessment of Phosphate-Buffered Saline (PBS) vehicle control- and doxorubicin-treated SH-SY5Y neuroblastoma cells using Agilent TapeStation. (**a**) High-Sensitivity RNA ScreenTape gel image showing the RNA ladder and total RNA profiles from vehicle control and doxorubicin-treated SH-SY5Y cells. (**b**) Representative electropherogram of the TapeStation RNA ladder demonstrating size standards from 25 to 6000 nucleotides. (**c**) Electropherogram of RNA extracted from PBS vehicle-treated SH-SY5Y cells. (**d**) Electropherogram of RNA extracted from doxorubicin-treated SH-SY5Y cells. The blue reference line serves as a calibration standard used for electrophoresis, while the orange arrow iddentifies the dominant ribosomal RNA band, indicative of RNA integrity and abundance in the analysed samples.

All RNA samples from vehicle control and treated neuroblastoma SH-SY5Y cells demonstrated good integrity, exhibiting RNA Integrity Number equivalent (RINe) values between 9.3 and 9.4 and 8.3 to 9.4, respectively (Figure 1). The 28S/18S ratios for both vehicle control and untreated SH-SY5Y RNA samples ranged from 2.4 to 2.8 and 2.3 to 2.6, respectively. DV200 values exceeding 80% indicated a high proportion of RNA fragments, >200 nucleotides appropriate for library preparation. RNA concentrations ranging between 8.49 and 23.6 ng/µL in treated SH-SY5Y RNA samples and concentrations ranging between 8.42 and 24.32 ng/µL in vehicle control SH-SY5Y RNA samples were quantified through the Qubit fluorometric system. This ensured enough mass for NEBNext Ultra II mRNA library preparation. Conjointly, all the metrics confirmed that the isolated RNA was of high integrity, purity, and fragment distribution standards that are required to conduct transcriptome sequencing conveniently.

### 2.2. KEGG (Kyoto Encyclopedia of Genes and Genomes) Pathway Enrichment Analysis and Gene Targets

**Figure 2:**
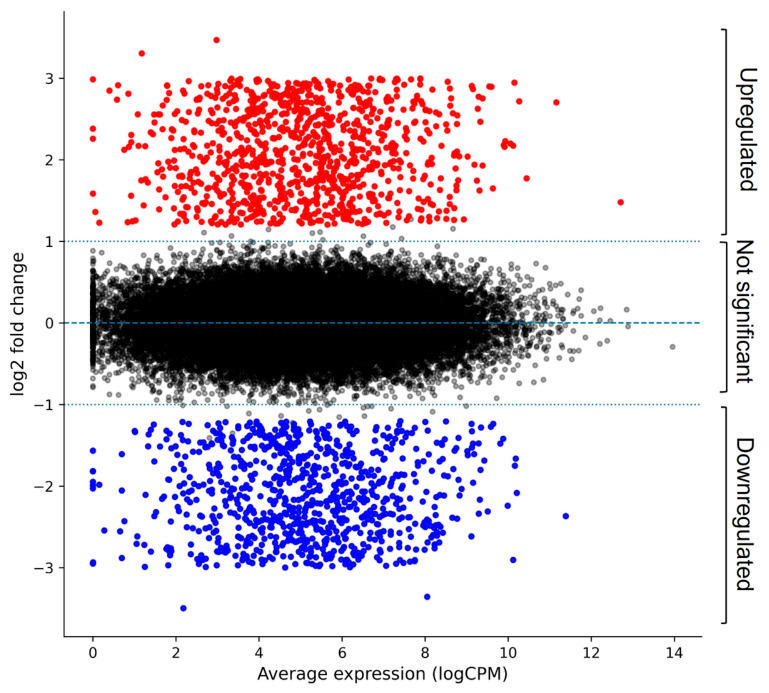
MA plot illustrates differential gene expression between vehicle control and doxorubicin-treated SH-SY5Y cells. Upregulated genes are shown in red, with downregulated genes in black, and insignificant genes in blue. Dashed horizontal lines indicate the thresholds for differential expression.

Analysis of the transcriptomic profiles revealed gene expression differences between untreated and doxorubicin-treated SH-SY5Y neuroblastoma cells. After filtering low-expression transcripts, a total of 37,856 genes with measurable expression were retained for analysis, and 1842 genes were significantly differentially expressed, including 1012 upregulated and 830 downregulated genes (Supplementary material Table S1). Global transcriptional changes across the dataset are visualized using an MA plot (Figure 2), illustrating the relationship between gene expression change (M) and average expression level (A). Candidate genes contributing to enriched biological pathways were further prioritized from the significantly differentially expressed gene set. Gene ranking was performed utilizing a composite prioritization strategy integrating differential expression magnitude (log_2_FC), statistical significance (adjusted *p*-value), and participation in significantly enriched KEGG (Kyoto Encyclopedia of Genes and Genomes) pathways. The top 20 prioritized genes contributing to pathway enrichment are presented in Table 1. The notable alterations in pathways associated with neurodegenerative ailments such as Alzheimer’s disease and conditions such as DNA damage, oxidative stress, and oncogenic signalling, with highly significant *p.adjust* values between 4.56 × 10^−9^ and 7.80 × 10^−6^, were unveiled through the KEGG pathway enrichment analysis. There are five key candidate targets that were identified through the integration of expression magnitude, pathway significance, and biological relevance. These targets are: *BRAF* (*p.adjust* = 4.56 × 10^−9^; Score = 4.966), *GSK3β* (*p.adjust* = 4.07 × 10^−7^; Score = 3.459), *PARP1* (*p.adjust* = 6.78 × 10^−8^; Score = 3.880), *BACE1* (*p.adjust* = 7.80 × 10^−6^; Score = 3.564), and *MAOB* (*p.adjust* = 7.89 × 10^−7^; Score = 4.032). A subset of genes, including *APP*, *PSEN1*, *PARP1*, *CDK5*, and *SNCA*, was robustly upregulated (log_2_FC values +0.824 to +1.396); these targets suggest the activation of amyloidogenic and stress-responsive pathways. Other pathway elements showed moderate to strong downregulation activity, such as the *VEGFA*, *EGFR*, *AKT1*, and *mTOR* factors. Conversely, most genes, particularly *FGFR3*, *BRAF*, *BACE1*, *GSK3β*, *NTRK1*, and *MAOB*, exhibited significant suppression; all genes had log_2_FC values between −1.240 and −2.146.

### 2.3. STRING Protein–Protein Interaction Network and Prediction of Binding Sites

**Figure 3:**
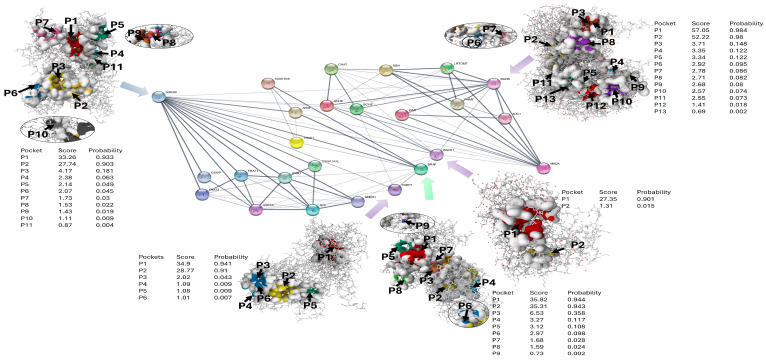
Predicted ligand-binding pockets (P1–Pn) of network-central hub proteins (BRAF, GSK3β, PARP-1, BACE1, and MAOB) identified using a machine-learning-based approach and mapped to STRING-derived interaction topology.

**Figure 4:**
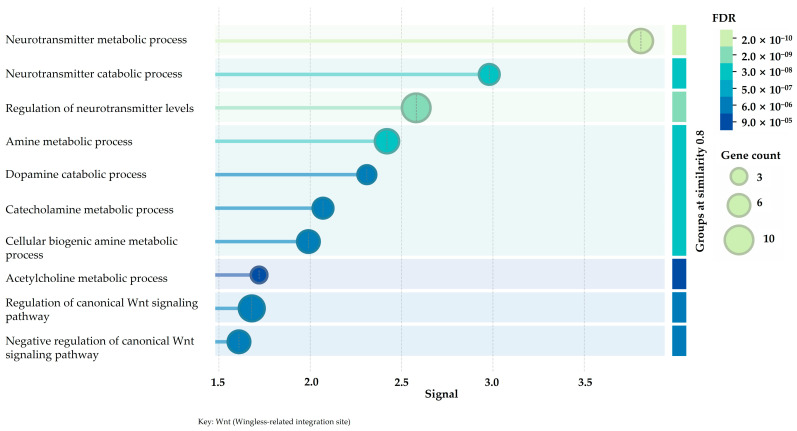
Functional enrichment dot plot illustrates biological processes linked with the stress-adaptive signalling network identified from transcriptomic analysis. Dot size indicating gene count and colour scale representing false discovery rate (FDR).

A dense interconnected network consisting of 20 proteins and 116 interactions was uncovered through STRING-based analysis of protein–protein interactions, with a clustering coefficient of 0.74, and an average node degree of 11.6 (Figure 3). These characteristics of a topological nature suggest significant functional unity and consistent information transfer and flow throughout the network. There are numerous genes that exhibited a significant degree of centrality, such as GSK3β, BRAF, AKT1, PARP1, and MAOB, marking them as hub nodes that connect and integrate various biological pathways. Target prioritization was informed not only by transcriptional direction but also by network centrality and pathway connectivity, as transcriptionally suppressed proteins might still play key regulatory roles in adaptive signalling networks. Notably, the identified hub proteins (BRAF, GSK3β, PARP1, BACE1, and MAOB) are functionally connected to the enriched biological processes presented in the functional enrichment dot plot (Figure 4), connecting kinase signalling, DNA repair, oxidative stress responses, and neurodegenerative pathway components within the stress-adaptive network.

There was a significant absence of poorly connected or isolated nodes that were observed, highlighting the functional unity of the chosen gene set. The functional enrichment analysis of the chosen gene set exhibited a significant overrepresentation of biological processes related to stress response, cell survival, neuronal signalling, and oncogenic control (Figure 4). The enrichment signals were robust false discovery rate (FDR) values, spanning 2.0 × 10^−10^ to 9.0 × 10^−5^, and involved roughly 3–10 genes per process, which is clearly indicative of coordinated pathway engagement rather than stochastic enrichment. Terms that were significantly enriched and converged more on kinase-mediated regulation and signal transduction, adding on pathways that govern the induction of apoptosis, oxidative stress responses, and functions related to neurodegeneration, aligning with the molecular environment of neuroblastoma cells facing genotoxic stress. Additionally, the prediction of binding sites unveiled druggable pockets of high confidence located in canonical catalytic or regulatory regions of all five targets, reinforcing their appropriateness for subsequent molecular docking and dynamic simulations (Figure 3).

### 2.4. Molecular Docking and Molecular Dynamics Simulations

**Figure 5:**
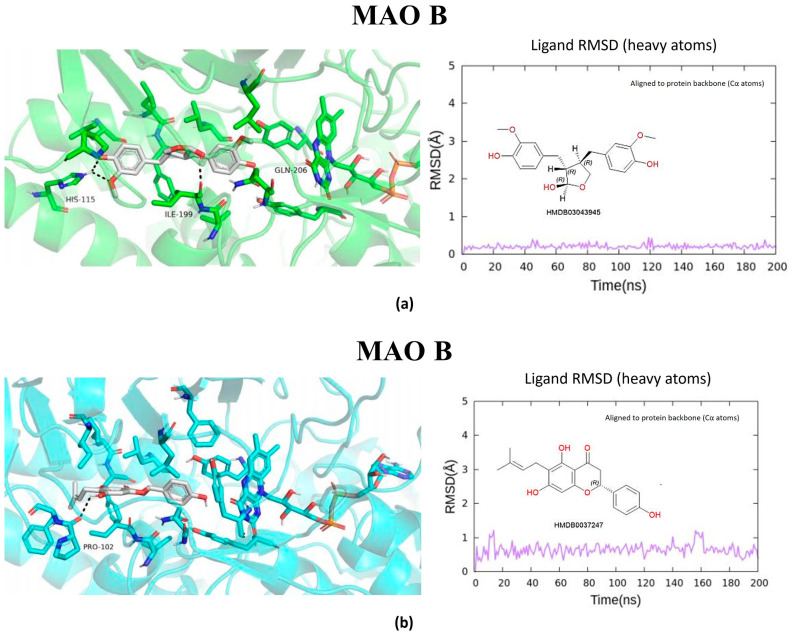

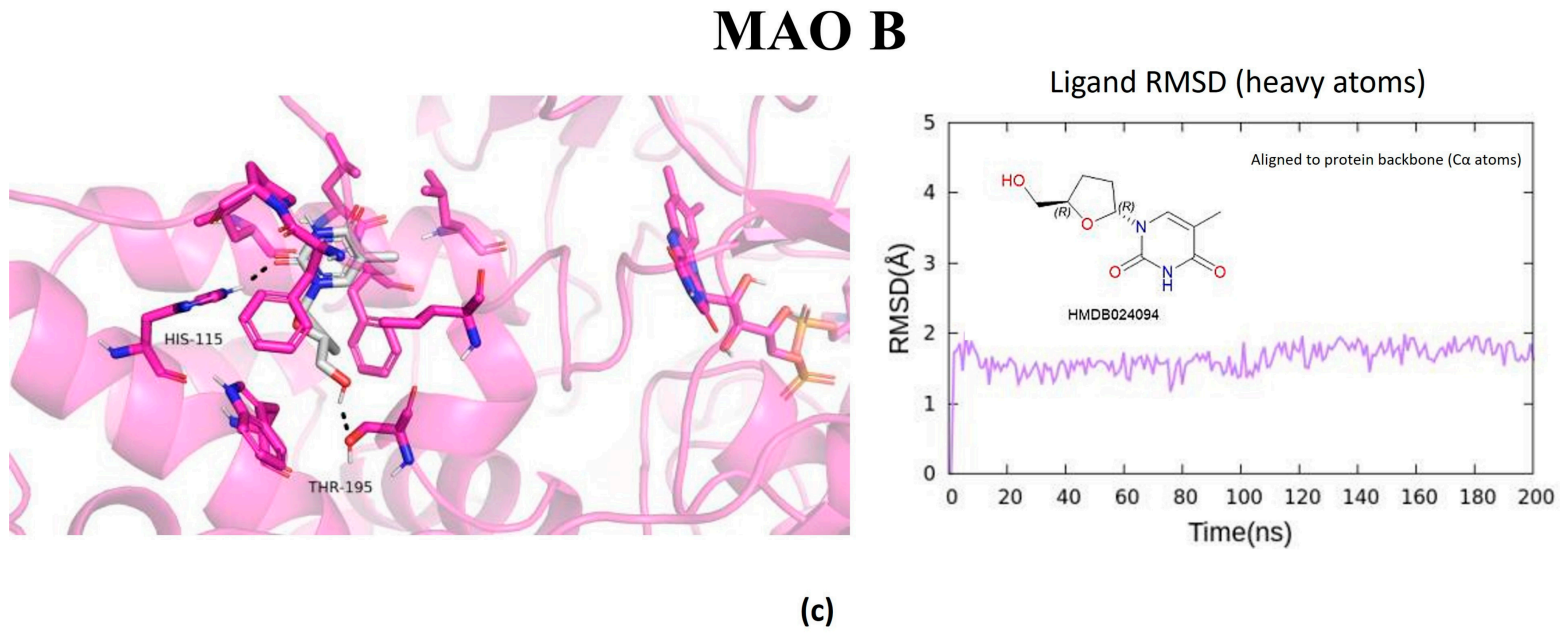
Molecular docking and molecular dynamics simulations of (**a**) Lactol, (**b**) 4′,5,7-Trihydroxy-6-prenylflavanone, and (**c**) 3′-Deoxythymidine binding with MAO B target. Ligand RMSD was calculated using all heavy atoms following alignment to the protein backbone (Cα atoms).

**Figure 6:**
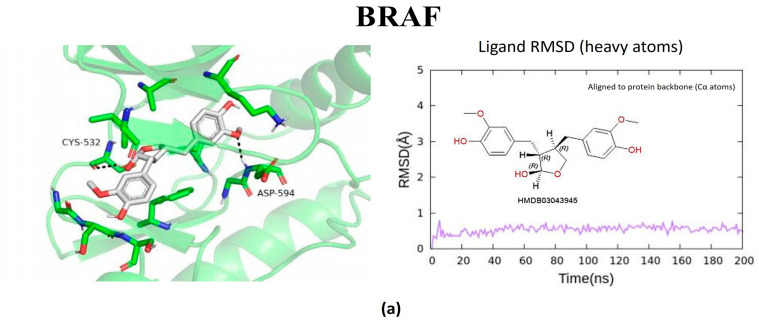

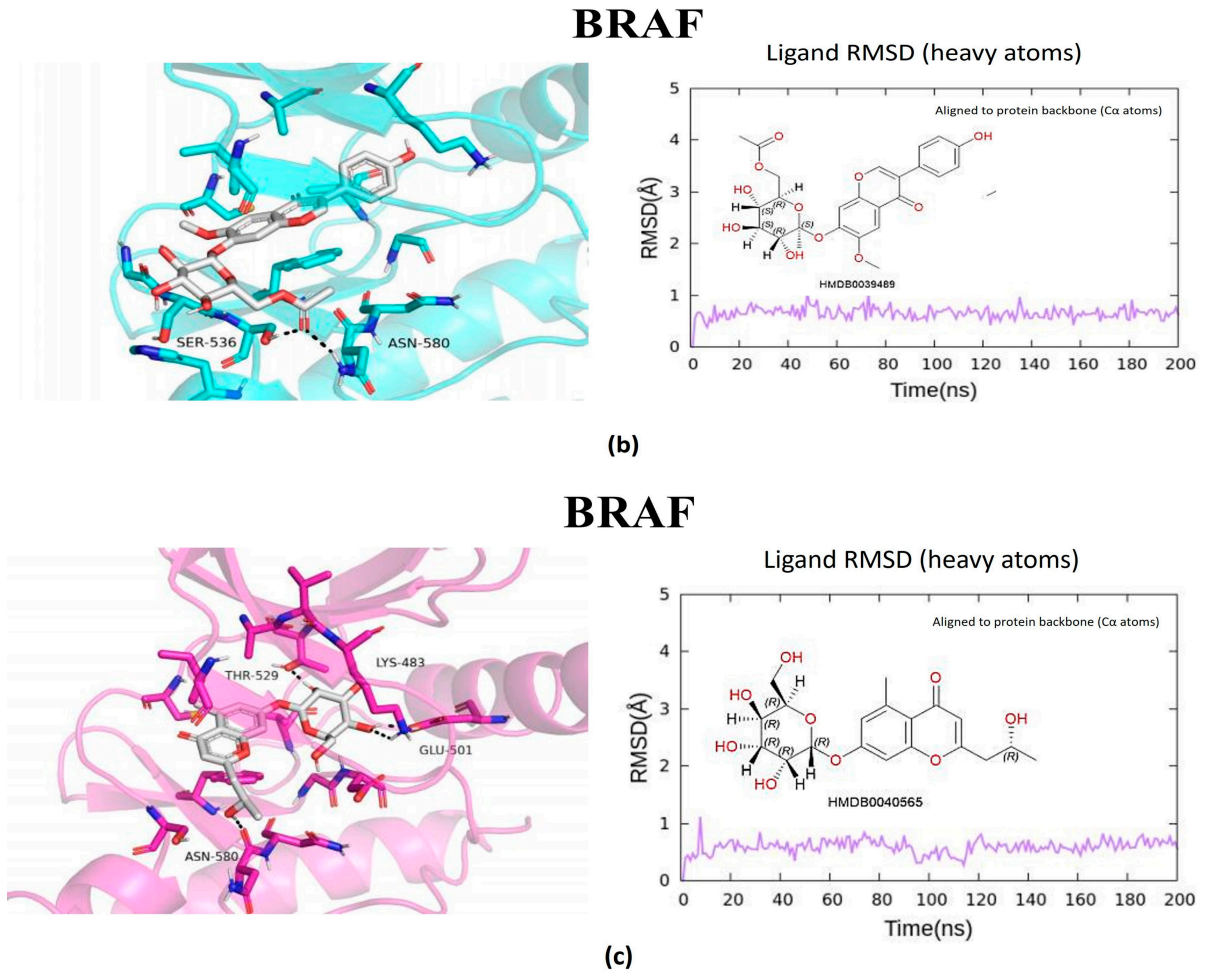
Molecular docking and molecular dynamics simulations of (**a**) Lactol, (**b**) 6″-O-Acetylglycitin, and (**c**) Aloesol 7-glucoside binding with BRAF target. Ligand RMSD was calculated using all heavy atoms following alignment to the protein backbone (Cα atoms).

**Figure 7:**
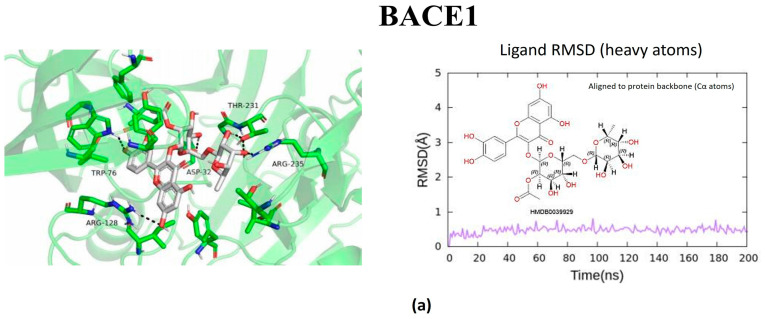

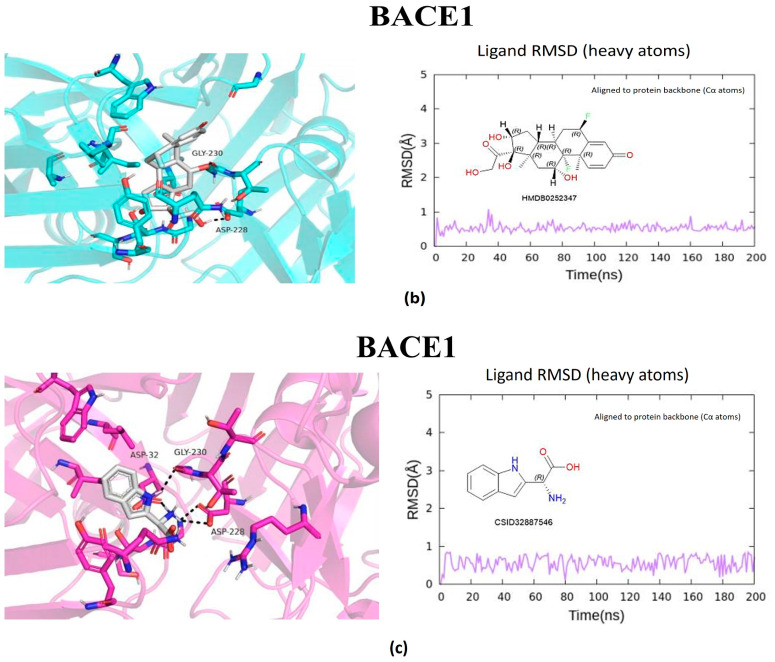
Molecular docking and molecular dynamics simulations of (**a**) 2″-O-Acetylrutin, (**b**) Fluocinolone, and (**c**) Amino(1H-indol-2-yl)acetic acid binding with BACE1 target. Ligand RMSD was calculated using all heavy atoms following alignment to the protein backbone (Cα atoms).

**Figure 8:**
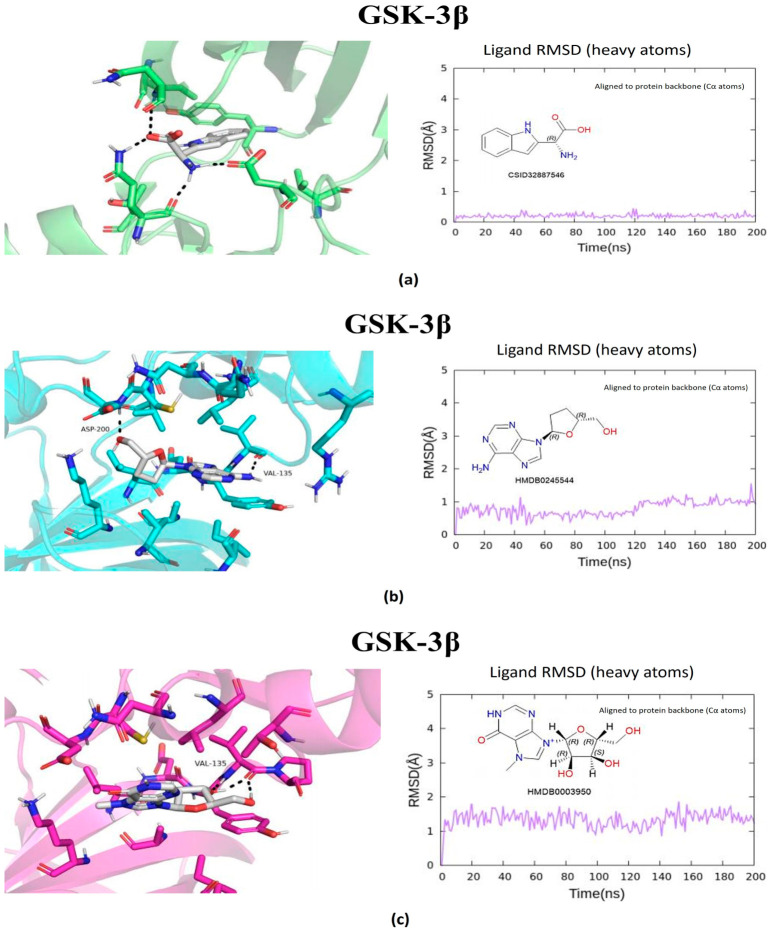
Molecular docking and molecular dynamics simulations of (**a**) Amino(1H-indol-2-yl)acetic acid, (**b**) 2′,3′-Dideoxyadenosine, and (**c**) 7-Methylinosine binding with GSK3β target. Ligand RMSD was calculated using all heavy atoms following alignment to the protein backbone (Cα atoms).

**Figure 9:**
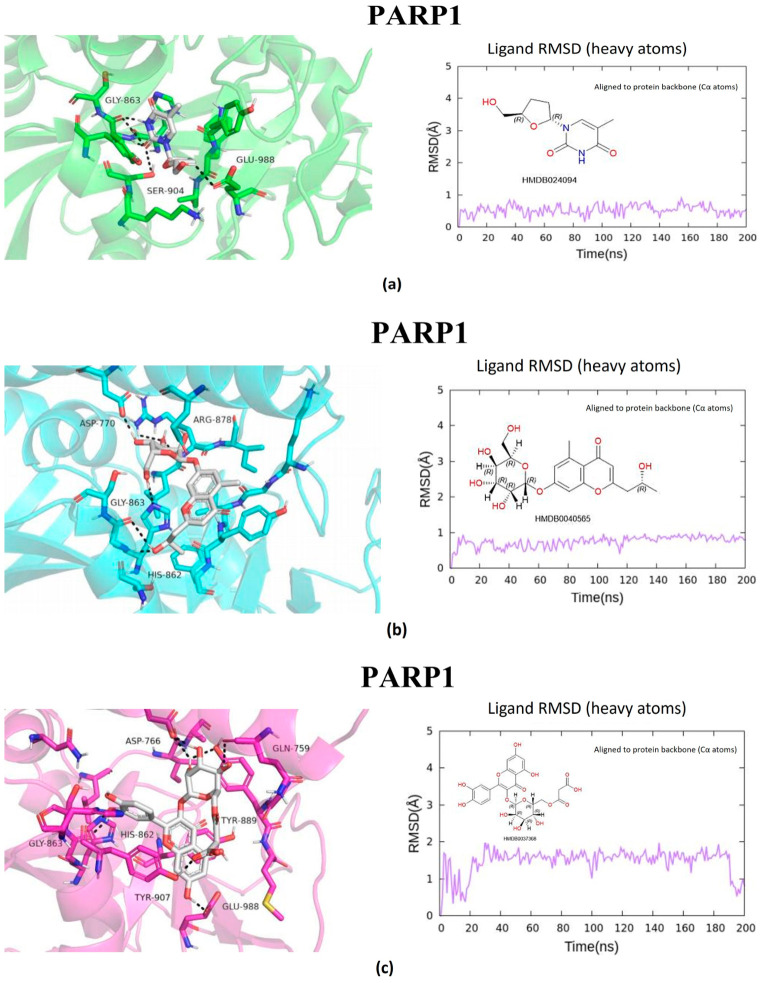
Molecular docking and molecular dynamics simulations of (**a**) 3′-Deoxythymidine, (**b**) Aloesol 7-glucoside, and (**c**) Quercetin 3-(6″-malonyl-glucoside) binding with PARP1 target. Ligand RMSD was calculated using all heavy atoms following alignment to the protein backbone (Cα atoms).

The metabolites evaluated in the docking and molecular dynamics simulations originated from metabolomic profiling of *L. javanica* and *A. calamus* extracts identified through Ultra-performance liquid chromatography–tandem mass spectrometry (UPLC-MS/MS) analysis, as described in Section 4.7. These metabolites were prioritized based on the statistical criteria of a VIP score greater than 1.5, *p*-value less than 0.05, FDR-adjusted *p* < 0.05 and a fold change (FC) greater than 2.0. Virtual screening of structures and 200 ns all-atom molecular dynamics simulations were conducted, and the end product was utilized to evaluate the binding stability and energetic preference of the selected plant metabolites against the prioritized hub proteins such as MAOB, BRAF, BACE1, GSK3β, and PARP1 (Figure 5, Figure 6, Figure 7, Figure 8 and Figure 9; Table 2, Supplementary Materials Table S2). Metabolite prioritization for molecular dynamics simulations and downstream experimental assessment was guided by an integrated framework including docking score, molecular dynamics stability, predicted binding free energy (ΔGbind) derived from MM-GBSA analysis, metabolomic significance, and predicted functional relevance to the stress-adaptive signalling pathways identified in the transcriptomic analysis.

RMSD trajectories of the protein backbone (Cα atoms) indicated that all protein–ligand complexes reached equilibrium during the early phase of the simulations and maintained structural stability throughout the 200 ns production run. Ligand RMSD, calculated using all heavy atoms following alignment to the protein backbone, exhibited minimal fluctuations, confirming stable ligand binding within the active sites of the respective targets (Supplementary Materials Figure S1). The three highest-ranked ligands were consistently attached to the specified binding sites during the trajectories of the simulations, which were used to establish hydrogen bonds, hydrophobic interactions, and π–π stacking. The three prioritized potential compounds were lactol, 4′,5,7-Trihydroxy-6-prenylflavanone, and 3′-Deoxythymidine against MAOB. Lactol exhibited the most favourable predicted binding free energy (ΔG_bind = −39.08 kcal/mol), with the ability to establish stable hydrogen bonds with H115, T201, and Q206, accompanied by π–π interactions involving residues of an aromatic nature, F99, F168, and Y326. Consistent interactions were exhibited by metabolites of 4′,5,7-Trihydroxy-6-prenylflavanone and 3′-Deoxythymidine, accompanied by binding free energies of −33.27 and −20.67 kcal/mol, respectively, outlining the strong engagement of the catalytic cavity belonging to MAOB. Consistent binding within the MAOB active site for 200 ns (Figure 5) was demonstrated by all three metabolites. The three prioritized potential compounds against *BRAF* were lactol, 6″-O-Acetylglycitin, and aloesol 7-glucoside. Molecular docking and dynamics simulations indicated a notably strong interaction of the *BRAF* kinase domain with the metabolites. The most favourable binding free energy was exhibited by lactol (ΔG_bind = −54.13 kcal/mol). This was additionally reinforced by hydrogen bonds with E501 and S536, hydrophobic interactions with W531 and L514, and π–π stacking with F583. Stability was exhibited by additional ligands such as 6″-O-Acetylglycitin (ΔG_bind = −47.38 kcal/mol) and aloesol 7-glucoside (ΔG_bind = −33.12 kcal/mol), occupying the ATP-binding area essential for kinase function (Figure 6).

The prioritized potential compounds against BACE1 were Amino(1H-indol-2-yl)acetic acid, 2″-O-acetylrutin, and fluocinolone. The compound with the best binding energy (ΔG_bind = −41.07 kcal/mol) was Amino(1H-indol-2-yl)acetic acid, establishing stable hydrogen bonds with the catalytic aspartates D32 and D228, along with π–π interactions with F108. Other compounds that exhibited stable binding sites were 2″-O-Acetylrutin (ΔG_bind = −35.25 kcal/mol) and fluocinolone (ΔG_bind = −28.96 kcal/mol). This suggests conducive interactions with the active site belonging to the aspartyl protease. The catalytic site of BACE1 showed strong and consistent interactions with multiple metabolites (Figure 7). The three prioritized potential compound against GSK3β were 7-Methylinosine, amino(1H-indol-2-yl)acetic acid, and 2′,3′-Dideoxyadenosine. The compound that demonstrated robust and stable binding to GSK3β (ΔG_bind = −47.38 kcal/mol) was amino(1H-indol-2-yl)acetic acid, aided by hydrogen bonds with D200 and hydrophobic interactions with I62, V70, and L188 in the kinase domain. The compounds 7-Methylinosine and 2′,3′-Dideoxyadenosine, with binding energies of ΔG_bind = −31.13 kcal/mol and ΔG_bind = −22.81 kcal/mol, respectively, also had predicted binding interaction, although there were slight conformational changes during the simulation analysis (Figure 8). Ultimately, the prioritized potential compounds against PARP1 were Quercetin 3-(6″-malonyl-glucoside), aloesol 7-glucoside, and 3′-Deoxythymidine. Quercetin 3-(6″-malonyl-glucoside) exhibited a highly favourable binding free energy (ΔG_bind = −46.03 kcal/mol), establishing extensive hydrogen bond networks with residues such as Y907, H862, and E988, as well as π–π interactions with Y896 and Y907. Aloesol 7-glucoside (ΔG_bind = −37.60 kcal/mol) and 3′-Deoxythymidine (ΔG_bind = −33.52 kcal/mol) also showed stable binding throughout the simulation period. Molecular dynamics simulations of PARP1 complexes revealed stable ligand interactions within the catalytic domain (Figure 9).

The top candidate compounds selected for in vitro testing based on molecular dynamics stability, MM-GBSA binding free energy calculations, metabolomic significance, and predicted functional relevance to the stress-adaptive signalling pathways were lactol for *MAOB* (ΔG_bind = −39.08 kcal/mol) and *BRAF* (ΔG_bind = −54.13 kcal/mol), Amino(1H-indol-2-yl)acetic acid for *BACE1* (ΔG_bind = −41.07 kcal/mol) and *GSK3β* (ΔG_bind = −47.38 kcal/mol), and Quercetin 3-(6″-malonyl-glucoside) for *PARP1* (ΔG_bind = −46.03 kcal/mol). These metabolites displayed favourable predicted binding interactions within the catalytic regions of the respective targets, reinforced by hydrogen bonding and π–π stacking interactions observed during the molecular dynamics simulations.

**Figure 10:**
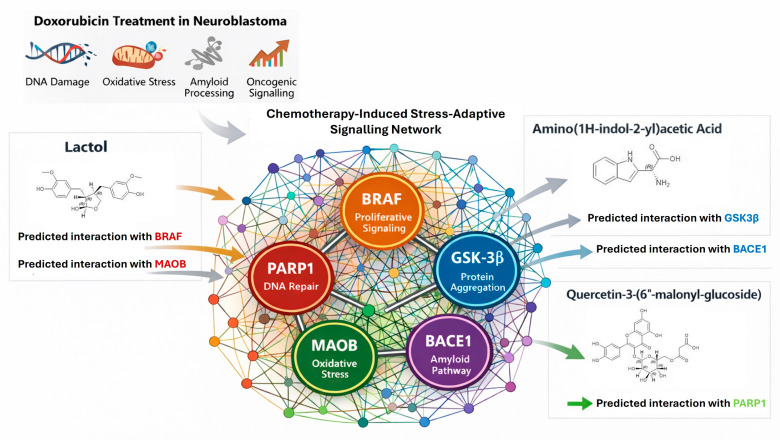
Representation of predicted interactions between selected plant metabolites and key proteins within stress-adaptive signalling pathways identified from transcriptomic analysis of doxorubicin-treated neuroblastoma cells.

Incorporation of transcriptomic, protein–protein interaction, and structure-based pharmacology information into a cohesive stress-adaptive network is summarized in Figure 10.

### 2.5. In Vitro Evaluation

#### Reduced Neuroblastoma Cell Viability Assay (CCK-8)

**Figure 11:**
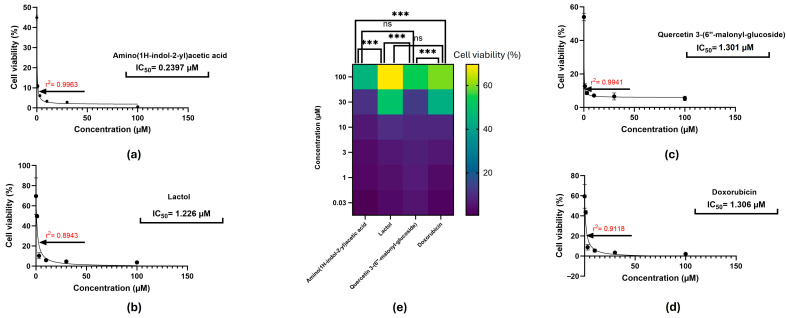
Evaluation of SH-SY5Y cell viability of selected compounds using the CCK-8 assay. SH-SY5Y neuroblastoma cells were treated with increasing concentrations (0.03–100 µM) of (**a**) Amino(1H-indol-2-yl)acetic acid, (**b**) Lactol, (**c**) Quercetin 3-(6″-malonyl-glucoside), and (**d**) Doxorubicin (positive control) for 24 h. (**e**) Heatmap of cell viability responses across all concentrations and compounds, revealing a clear concentration-dependent reduction in viability. The colour scale represents percentage cell viability, where darker purple indicates lower viability and yellow indicates higher viability. Key: *** indicates a statistically significant difference; ns indicates no statistically significant difference.

The Cell Counting Kit-8 (CCK-8) assay was utilized to evaluate the effects of the selected compounds, Amino(1H-indol-2-yl)acetic acid, lactol, and Quercetin 3-(6″-malonyl-glucoside), and the positive control doxorubicin, on SH-SY5Y neuroblastoma cells. The cells were subjected to each compound at increasing concentrations (0.03–100 µM) for a period of 24 h, revealing dose-dependent decreases in cell viability, which were observed (Figure 11). The compound exhibiting the most potent anti-neuroblastoma effect was Amino(1H-indol-2-yl)acetic acid, demonstrating an IC_50_ value of 0.2397 µM (r^2^ = 0.9963) and a cell viability of 0.56 ± 0.43% at 100 µM, which was, importantly, a marked decrease to 44.95 ± 1.04% even at 0.03 µM. A comparable potency was demonstrated by the compound lactol with an IC_50_ of 1.226 µM (r^2^ = 0.8943), and cell viability percentages of 3.67 ± 0.029% and 69.70 ± 1.08% at concentrations of 100 µM and 0.03 µM, respectively. Moderate efficacy with an IC_50_ of 1.301 µM (r^2^ = 0.9941), leading to 5.36 ± 1.34% and 54.02 ± 2.27% viability at 100 µM and 0.03 µM, respectively, was exhibited by Quercetin 3-(6″-malonyl-glucoside). Doxorubicin, the reference drug, highlighted an IC_50_ of 1.306 µM (r^2^ = 0.9118), exhibiting 2.10 ± 0.57% viability at 100 µM and 59.49 ± 1.81% viability at 0.03 µM. Figure 11 displays a heatmap outlining the distinct concentration-dependent decrease in cell viability. Groups combining Amino(1H-indol-2-yl)acetic acid and Quercetin 3-(6″-malonyl-glucoside) were observed to have significant differences in comparison to lactol and doxorubicin (*p* < 0.0001). This confirms the different effects of various classes of compounds. Furthermore, the compound type and concentration relationship, particularly in comparing Amino(1H-indol-2-yl)acetic acid and Quercetin 3-(6″-malonyl-glucoside) with lactol and doxorubicin, was noteworthy in detecting and emphasizing different dose–response behaviours.

## 3. Discussion

The clinical intractability of neuroblastoma is increasingly attributed to integrated networks of survival that encompass kinase signalling, DNA damage repair, mitochondrial stress, and adaptive programmes rather than isolated oncogenes [18]. This enables tumours to exhibit systemic plasticity that can withstand chemotherapeutic treatments through a multi-pathway coordination rather than a singular pathway dependence [19,20]. In this context, beyond their role as DNA-intercalating drugs, cytotoxic agents such as doxorubicin also induce extensive transcriptional and signalling reconfiguration, specifically engaging cell-cycle regulators, stress-activated kinases, and DNA repair pathways [20,21,22,23]. Exposure in SH-SY5Y neuroblastoma cells elicits a dual neuro-oncogenic phenotypical state of stress defined by simultaneous activation of amyloidogenic processing, oxidative stress responses, kinome signalling, and DNA repair mechanisms, illustrating a convergence of neurodegenerative and neoplastic stress responses under prolonged genotoxicity [23,24,25,26]. Comparable integrated adaptive states have been documented and observed across various therapy-resistant cancer malignancies, whereby surviving cells achieve homeostatic stability through the coordination of DNA repair, mitochondrial redox regulation, and pro-survival signalling cascades, thereby maintaining cell viability in spite of prolonged drug exposure [27,28]. Transcriptomic profiling was conducted 48 h post-doxorubicin exposure to isolate this adaptive response rather than acute damage sensing alone [29,30]. This interval aligns with time-resolved transcriptomic data to coincide with the peak systemic network reconfiguration and the activation of stress repair and survival programmes, which typically occur 24 and 72 h following treatment [29,30].

Our transcriptomic profiling reveals a significant upregulation of genes associated with Alzheimer’s pathology (*APP*, *PSEN1*, and *BACE1*), synaptic modulation (*MAPT*, *CDK5*, *SNCA*), canonical oncogenic signalling (*BRAF*, *AKT1*, *MTOR*, *EGFR*, *FGFR3*), and oxidative stress (*SOD2*, *MAOB*), which was induced by doxorubicin [31]. Upregulation of *APP*, *PSEN1*, *CDK5*, and *SNCA* may reflect activation of amyloidogenic and neurodegenerative stress pathways that are increasingly recognized in neural-lineage tumours such as neuroblastoma under genotoxic stress [31,32]. This dual molecular profile aligns with reports that stress induced by genotoxicity may trigger amyloidogenic and mitochondrial impairment in neural lineage cells [33]. Furthermore, stress-induced modulation of amyloid precursor metabolism has been a critical driver of tumour adaptation and metastasis in cancer [34,35,36]. The simultaneous induction of DNA repair and survival-related kinases signifies a systemic response to stress that traditional monotherapies may paradoxically strengthen. Through the analysis of network biology, this transcriptional heterogeneity has been refined into a central regulatory hub comprising BRAF, GSK3β, PARP1, MAOB, and BACE1. This highlights an integrated module instead of signalling nodes that are isolated. These findings corroborate a model whereby therapeutic stress co-opts conservation modules common to both neurodegeneration and oncogenesis, yielding a state of network addiction that may represent a potential vulnerability for future therapeutic investigation [13].

Utilizing a structure-based approach, plant-derived metabolites were identified as candidate ligands with predicted multi-target interaction profiles within the stress-adaptive signalling network. Notably, lactol displayed predicted binding interaction to MAOB and BRAF, suggesting potential binding compatibility with both targets in the disruption of MAPK-mediated proliferation and mitochondrial ROS generation [37,38,39]. Although the cytotoxic activity of lactol was assessed independently in this study, future investigations incorporating antioxidant co-treatment experiments, such as N-acetyl cysteine, may assist to clarify whether the observed cytotoxic response is mediated through ROS-dependent mechanisms. Nonetheless, this therapeutic strategy mirrors dual targeting approaches in Parkinson’s disease models and melanoma [40]. The structural integrity of the complex between aromatic hydrogen bonding and π–π stacking interactions was assessed through 200 ns molecular dynamics, together with the predicted free energies, which display robust molecular engagement, which is indicative of established mechanisms of effective kinase and inhibitors of flavoenzyme [41]. The compound Amino(1H-indol-2-yl)acetic acid exhibited high predicted binding interaction to BACE1 and GSK3β, key enzymes involved in amyloid generation and survival signalling. Given that GSK3β serves as a critical nexus for the Wnt, apoptosis, and signalling of β-catenin, its pharmacological inhibition has been reported to show enhanced efficacy of chemotherapy in a wide array of cancers [42,43]. The pharmacological inhibition of BACE1 decreases the processing of toxic APP, and the combined blockade of BACE1 or GSK3β has demonstrated the cooperative therapeutic impact in Alzheimer’s disease models and cancers characterized by neural-lineage signatures [44]. Structural characterization identified Quercetin-3-(6″-malonyl-glucoside) as a robustly engaged ligand for PARP1, which is a critical regulator of DNA repair pathways, and its inhibition has been clinically leveraged in tumours that are BRCA-deficient [45]. To contextualize the predicted binding interaction achieved in this study, the docking results were compared with reported structurally characterized inhibitors of the respective target proteins. For example, clinically relevant BRAF inhibitors including vemurafenib and dabrafenib typically exhibit strong binding connections within the ATP-binding pocket of the kinase domain [46]. Similarly, MAOB inhibitors, for instance, selegiline, interact with residues within the catalytic cavity of the flavin-containing amine oxidase [47]. BACE1 inhibitors such as verubecestat are known to create hydrogen bonds with catalytic aspartate residues (Asp32 and Asp228) [48], while GSK3β inhibitors, for instance, tideglusib, target the ATP-binding region of the kinase [49]. Furthermore, PARP1 inhibitors such as olaparib interact with the catalytic NAD^+^ binding site during hydrogen bonding and aromatic stacking interactions [45]. Through occupancy of the PARP1 catalytic site, quercetin derivatives replicate essential pharmacophores that have been approved as inhibitors of PARP, such as olaparib, but utilizing a natural compound backbone underscores the viability of ‘nature-inspired’ chemosensitisation [50,51].

In the SH-SY5Y neuroblastoma model, all three metabolites significantly suppressed cellular viability, with doxorubicin’s potency exceeded by Amino(1H-indol-2-yl)acetic acid. Differences between predicted docking affinities and experimental IC_50_ values likely reflect the fact that molecular docking evaluates structural binding compatibility, whereas cellular assays are influenced by additional factors, for instance, membrane permeability, metabolic stability, and intracellular target accessibility. The CCK-8 assay offers quantitative assessment of relative cell viability based on metabolic activity; it does not differentiate between cytostatic and cytotoxic effects, nor does it directly quantify apoptosis, clonogenic survival, or long-term proliferative capacity [52,53,54]. Consequently, the observed reduction in viability should be interpreted as preliminary evidence of cytotoxic or anti-proliferative potential. Further mechanistic validation through apoptosis assays, cell-cycle analysis, clonogenic survival studies, assessment across additional neuroblastoma models, and evaluation of selectivity in non-tumourigenic cells will be needed to comprehensively establish anti-neuroblastoma efficacy. These observations are consistent with the predicted multi-target interaction profiles of the metabolites [55,56]; although, the present study did not directly evaluate network-level perturbations or synergistic multi-target effects. This network-centric interpretation of pharmacological activity is grounded in systems of a biological nature, which characterize states of disease as an emergent phenomenon of complex interconnected molecular networks rather than isolated linear pathways [57]. In accordance with this network addiction model, doxorubicin treatment precipitates a dual stress state in the SH-SY5Y neuroblastoma cells defined by the synchronous activation of DNA damage repair (PARP1), oxidative stress buffering (MAOB), amyloidogenic processing (BACE1), neurodegenerative kinase signalling (GSK3β), and oncogenic proliferation (BRAF). The integration of transcriptomic profiling and protein–protein interaction demonstrates that the pathways converge into an interconnected stress-adaptive network characterized by high hub centrality as depicted in Figure 10. In silico analysis, utilizing structure-based docking and molecular dynamics simulations, confirms that the three bioactive plant metabolites possess polypharmacological profiles precisely aligned with the tumor’s network topology where lactol targets BRAF and MAOB, the dual targeting of BACE1 and GSK3β by Amino(1H-indol-2-yl)acetic acid targets, and Quercetin-3-(6″-malonyl-glucoside) targets PARP1. The predicted multi-target binding profiles of these metabolites suggest possible interactions with components of the chemotherapy-associated stress-adaptive signalling network identified in the transcriptomic analysis. These predicted multi-target interactions provide a potential mechanistic explanation for the observed reduction in neuroblastoma cell viability, although direct experimental validation of network-level perturbations was not performed.

Collectively, the present study provides an exploratory framework connecting transcriptomic profiling, protein–protein interaction analysis, and structure-based computational screening to reveal plant-derived metabolites with potential relevance to stress-adaptive signalling pathways in neuroblastoma cells. However, various limitations should be acknowledged. The findings are based on a single neuroblastoma cell model and a single viability assay, and the predicted protein–ligand interactions are derived from computational docking and molecular dynamics simulations instead of direct biochemical validation. An additional limitation of the present study is the absence of comparative molecular dynamics simulations using co-crystallized ligands as positive controls. Furthermore, it is important to note that natural products frequently exhibit polypharmacological behaviour and might interact with multiple molecular targets beyond those evaluated in the present analysis [58,59]. Therefore, the docking and molecular dynamics simulations presented herein should be interpreted as predictions of potential binding compatibility rather than confirmation of selective target engagement. The observed effects should be interpreted as preliminary indications of cytotoxic or anti-proliferative potential. Future studies incorporating additional neuroblastoma models, orthogonal functional assays, and in vivo experimental systems will be required to validate the biological activity, target engagement, and therapeutic relevance of the identified metabolites. Moreover, studies incorporating post-treatment recovery models might further clarify whether the identified stress-adaptive transcriptional programmes represent transient responses or stable reprogramming events following chemotherapy exposure. Nevertheless, the metabolites characterized from *L. javanica* and *A. calamus* provide structurally diverse candidates that may serve as starting points for further investigation of multi-target interactions within stress-adaptive signalling pathways.

## 4. Materials and Methods

### 4.1. Reagents

Dulbecco’s Modified Eagle’s Medium (DMEM) supplemented with heat-inactivated fetal bovine serum was sourced from Gibco (New York, NY, USA), Phosphate-Buffered Saline (PBS), Trypsin-EDTA, and dimethyl sulfoxide (DMSO) were acquired from Sigma-Aldrich (St. Louis, MO, USA), and Doxorubicin hydrochloride was procured from Abcam (Cambridge, UK).

### 4.2. Cell Culture and Treatment

SH-SY5Y human neuroblastoma cells procured from Cellonex™, Johannesburg, South Africa, were cultured in DMEM supplemented with 10% (*v*/*v*) heat-inactivated fetal bovine serum and sustained at a temperature of 37 °C within a humidified incubator that maintained a 5% CO_2_ atmosphere. Cells were seeded in 6-well plates at 2 × 10^5^ cells per well and permitted to adhere overnight, to be used for transcriptomic analysis. Subsequently, the cells were subjected to treatment in triplicate for 48 h, with the treatment solution prepared and dissolved in sterile phosphate-buffered saline (PBS). Transcriptomic profiling was conducted 48 h post-exposure to 1 µM doxorubicin, which was administered as doxorubicin hydrochloride dissolved in sterile PBS. This concentration of doxorubicin was selected because it is regularly used in neuroblastoma cell models to induce measurable stress and cytotoxic responses while maintaining sufficient viable cells for transcriptomic analysis. The 48 h time point was chosen to capture stabilized transcriptional responses linked with chemotherapy-induced stress adaptation, whereas earlier time points typically display acute DNA-damage signalling events following doxorubicin exposure [29,30]. The vehicle control consisted of culture medium containing sterile PBS at a volume equivalent to that used for doxorubicin dilution. Upon completion of the treatment, the culture medium was aspirated and the cells were washed with PBS and then lysed in RLT buffer supplemented with 1% β-mercaptoethanol (QIAGEN, Hilden, Germany) immediately thereafter to facilitate RNA extraction.

### 4.3. Extraction of RNA, Quality Control, and Preparation of RNA-Seq Libraries

SH-SY5Y cell lysates were used to isolate total RNA with the RNeasy Mini Kit (QIAGEN, Hilden, Germany, Cat. No. 74104) following the manufacturer’s protocol, which included on-column DNase digestion to eliminate genomic DNA. The amount of RNA was quantified with a Qubit 4 Fluorometer (Thermo Fisher Scientific, Waltham, MA, USA), and the RNA integrity number (RIN) was assessed on an Agilent 4200 TapeStation (Agilent Technologies, Santa Clara, CA, USA) utilizing High-Sensitivity RNA ScreenTape to acquire RINe values. Samples were deemed acceptable for poly(A)-enriched RNA-seq library preparation only if they had RINe ≥ 7, a 28S/18S ratio > 2.0, and DV200 > 70%.

For every sample, 200 ng–1 µg of total RNA free of DNA was utilized as input for the NEBNext Ultra II RNA Library Prep Kit (New England Biolabs, Ipswich, MA, USA, Cat. No. E7775L) for Illumina, along with the NEBNext Poly(A) mRNA Magnetic Isolation Module (New England Biolabs, Ipswich, MA, USA, Cat. No. E7490L), adhering to the instructions provided by the manufacturer. Additionally, oligo(dT) magnetic beads were used to capture poly(A)^+^ RNA, which was then washed and eluted, followed by fragmentation through heat and synthesis of the first-strand cDNA with random primers. Double-stranded cDNA was obtained from the generation of second-strand cDNA, followed by end-repair, A-tailing, and ligation to NEBNext adaptors. Following purification and size selection with NEBNext Sample Purification Beads, adaptor-ligated fragments underwent amplification by PCR utilizing NEBNext index primers. The final libraries were purified using magnetic beads and were evaluated on an Agilent Bioanalyzer DNA chip to verify the anticipated fragment size distribution (~300 bp) and quantified before sequencing on an Illumina NextSeq 2000 platform. Raw sequencing reads were firstly demultiplexed with the NextSeq 2000′s onboard DRAGEN v4.2.7 software and followed by preliminary quality assessment, which included quality profiling per base, screening for adaptor contamination, and GC-content analysis. RNA sequencing was achieved using three biological replicates per condition (vehicle control and doxorubicin-treated SH-SY5Y cells). Raw sequencing reads were subjected to quality assessment using FastQ version 0.11.9, and adapter sequences as well as low-quality bases were trimmed using Trimmomatic version 0.39. Reads with a Phred quality score below Q30 were trimmed or removed during quality filtering prior to downstream analysis. High-quality reads were aligned to the human reference genome (GRCh38) using HISAT2 version 2.2.1, and alignment quality was assessed by calculating mapping statistics, including total read depth, overall mapping rate, and uniquely mapped reads. Only high-quality aligned reads were retained for differential expression analysis. Sequencing produced an average of 20–30 million paired-end reads per sample, with >90% of bases exceeding Q30 and >85% uniquely mapped reads across all libraries.

Clean, high-quality reads were mapped to the *Homo sapiens* reference genome utilizing a standardized RNA-seq alignment process. Gene-level read counts were obtained from aligned sequencing reads, and normalized transcript abundance was additionally calculated as fragments per kilobase of transcript per million mapped reads (FPKM). Raw count matrices were utilized for differential expression analysis, while FPKM values were used for descriptive reporting of gene expression levels.

### 4.4. Analysis of Differential Gene Expression and Enrichment

Differential expression analysis was performed utilizing raw gene count matrices obtained from RNA-seq alignment. Counts were normalized using the voom transformation implemented in the limma package. The analysis was conducted using the limma-voom pipeline, which applies empirical Bayes-moderated linear modelling to estimate gene-wise differential expression. *p*-values obtained from gene-wise statistical testing were adjusted using the Benjamini–Hochberg procedure and the resulting adjusted *p*-values were reported as false discovery rate (FDR) values. Genes satisfying |log_2_ fold change| ≥ 0.5 and an adjusted *p*-value (FDR) < 0.05 were considered significantly differentially expressed. Global transcriptional patterns were visualized using volcano plots to illustrate the magnitude and statistical significance of differential expression across the transcriptome. The identified differentially expressed genes (DEGs) were subsequently subjected to Kyoto Encyclopedia of Genes and Genomes (KEGG) pathway enrichment analysis to determine biological pathways significantly altered following doxorubicin exposure.

### 4.5. Data Consolidation, Evaluation, and Target Ranking

Following differential expression analysis, genes satisfying the statistical criteria (|log_2_FC| ≥ 0.5 and adjusted *p*-value (FDR) < 0.05) were retained for pathway enrichment analysis. The resulting list of significantly differentially expressed genes (DEGs) was subjected to KEGG pathway enrichment analysis to identify biological pathways significantly altered following doxorubicin treatment. Genes contributing to enriched KEGG pathways were prioritized using a composite ranking strategy integrating pathway significance, gene expression magnitude, and pathway relevance. A composite prioritization score was calculated for each gene using the following equation

$$\mathrm{Score}=0.5\times [-{log}_{10}(p.adjust)]+0.3\times {log}_{2}(\mathrm{FPKM}+1)+0.2\times \mathrm{Pathway}\;\mathrm{Relevance}.$$

where *p.adjust* represents the Benjamini–Hochberg adjusted *p*-value (FDR) derived from KEGG pathway enrichment analysis, FPKM represents gene expression abundance, and Pathway Relevance denotes pathway membership weighting (1.0 for genes belonging to the top five enriched KEGG pathways and 0.5 for genes present in other significantly enriched pathways).

Genes were ranked from highest to lowest composite score, and the top 20 highest-ranking genes were selected for downstream structural and network analysis. The ultimate selection of the five protein targets for molecular docking and molecular dynamics simulations was based on a multi-criteria prioritization strategy. Candidate genes were required to simultaneously satisfy the statistically significant differential expression (adjusted *p*-value (FDR) < 0.05 and |log_2_FC| ≥ 0.5), high composite prioritization score ranking (within the top 20 genes), and documented functional involvement in stress-adaptive signalling pathways relevant to neuroblastoma biology, as supported by KEGG pathway enrichment results and literature-based biological annotation.

### 4.6. STRING Network for Protein–Protein Interactions and Prediction of Binding Sites

A protein–protein interaction (PPI) network was created utilizing STRING database version 12.5. (http://string-db.org/; accessed on 12 November 2025). The input gene set comprised the five prioritized targets (*BRAF*, *PARP1*, *BACE1*, *GSK3β*, and *MAOB*) identified through composite score ranking and functional relevance analysis (FDR < 0.05; log_2_FC ≥ 0.5). *Homo sapiens* was specified as the reference organism.

All STRING evidence channels included experimental data, curated databases, text mining, co-expression, gene neighbourhood, gene fusion, and co-occurrence to preserve interaction completeness. Network expansion enabled inclusion of first-shell interactors for contextualization of the prioritized targets within a broader stress-adaptive interaction landscape. A high-confidence interaction threshold (combined score ≥ 0.700) was implemented. The resulting interaction network was evaluated to confirm functional connectivity and biological coherence among the selected stress-adaptive targets. The generated interaction network was exported from STRING in TSV format for subsequent topological analysis and downstream visualization using Cytoscape (version 3.10.4; accessed on 12 November 2025).

For structural modelling, high-resolution human crystal structures (resolution ≤ 2.5 Å) with complete catalytic domains were retrieved from the Protein Data Bank (https://www.rcsb.org/; accessed on 18 November 2025). Ligand-binding sites were predicted using P2Rank (version 2.5; retrieved on 14 November 2025) via the PrankWeb interface. Predicted binding pockets were prioritized based on probability score and spatial correspondence with canonical catalytic residues, and high-confidence catalytic-region pockets were retained for downstream molecular docking and molecular dynamics simulations studies. These catalytic pockets correspond to experimentally characterized functional domains reliable for enzymatic activity, comprising kinase ATP-binding sites and catalytic enzyme cavities where ligand binding modulates protein function [60,61].

### 4.7. Selection and Preparation of Metabolite-Based Libraries

A compound-based library was generated from significant metabolites identified in extracts of *L. javanica* and *A. calamus*, utilizing data from both ESI^+^ modes. The methodology outlined by Motshudi and colleagues [62] provided the foundation for the process of identifying the collection of bioactive metabolites.

The sample preparation comprised a freeze-drying process (lyophilization) of *A. calamus* and *L. javanica* leaf samples collected from Hartbeespoort in the North-West Province of South Africa (25.7236° S, 27.9653° E). Subsequently the samples were crushed in a 5 mL homogenizing tube at 30 Hz with four 5 mm metal balls in an MM 400 mill mixer (Thermo Fisher Scientific, Waltham, MA, USA)**.** The subsequent product for each sample was added with 80% methanol (Sigma-Aldrich (St. Louis, MO, USA)) at a volume of 800 µL. The samples were vortexed for 30 s and sonicated afterward for 30 min at 4 °C. The samples were subsequently stored for an hour at a temperature of -20 °C. Each sample was centrifuged thereafter at 12,000 rpm, at a temperature of 4 °C for 5 min. A total of 200 µL of each supernatant from the respective samples, along with an internal standard (5 μL) of DL-o-Chlorophenylalanine from Sigma-Aldrich (St. Louis, MO, USA) (0.14 mg/mL), was added to each sample. The vials were then used for liquid chromatography–mass spectrometry (LC-MS) analysis. QC samples were used to demonstrate the stability of the LC-MS system.

An ACQUITY UPLC HSS T3 column (100 mm × 2.1 mm, 1.8 µm) (Thermo Fisher Scientific, Waltham, MA, USA) coupled with the main system of the Ultimate 3000 LC and a Q Exactive mass spectrometer (Thermo Fisher Scientific, Waltham, MA, USA) was employed for the untargeted UPLC-MS/MS analysis. The mobile phase system consisted of (A) 0.05% formic acid water from Sigma-Aldrich (St. Louis, MO, USA) and (B) acetonitrile from Sigma-Aldrich (St. Louis, MO, USA), with the gradient elution of 0–1 min, 95% A; 1–12 min, 5–95% A; 12–13.5 min, 5% A; 13.5–13.6 min, 5–95% A; and 13.6–16 min, 95% A. The flow rate was maintained at 0.3 mL min^−1^, with the column and autosampler temperatures set to and maintained at 40 °C. Electrospray ionization in positive mode (ESI^+^) was employed with mass spectrometry parameters of heater temperature 300 °C; sheath gas flow rate, 45 arb; auxiliary gas flow rate, 15 arb; sweep gas flow rate, 1 arb; spray voltage, 3.0 kV; capillary temperature, 350 °C; S-Lens RF level, 30%. Metabolite identification was achieved at a putative level (MSI level 2) employing accurate mass measurements and MS/MS fragmentation data. Metabolites were annotated by matching experimental spectra against the Human Metabolome Database (www.hmdb.ca), MassBank (https://massbank.eu/MassBank/), ChemSpider (www.chemspider.com), all accessed 19 August 2024. Moreover, manual evaluation of retention time consistency, isotope patterns, and MS/MS fragmentation spectra was done. Cross-validation across databases was done to improve annotation confidence. Features detected in the ESI^+^ mode were cross-checked for chromatographic and spectral reliability and combined where appropriate. Redundant ions and adducts were resolved applying mass accuracy criteria (<5 ppm), retention-time coherence, signal intensity, and % RSD across technical replicates to reduce duplication or erroneous assignments. SIMCA-P software (version 14.1) and Metaboanalyst version 6.0, https://www.metaboanalyst.ca (accessed on 12 August 2025) were used for univariate and multivariate analysis.

Metabolites selected for molecular docking, molecular dynamics simulations, and in vitro analyses were based on the statistical criteria of variable importance in projection (VIP) score greater than 1.5, a *p*-value less than 0.05, FDR-adjusted *p* < 0.05 and a fold change (FC) greater than 2.0. Metabolites selected for further in silico and in vitro assessment are shown in Table 2, also found in Supplementary Materials Table S3.

### 4.8. In Silico Studies

#### 4.8.1. Ligand Preparation

Ligand structures retrieved from HMDB and ChemSpider were prepared using AutoDockTools v1.5.7 [63]. Protonation states were assigned at physiological pH (7.4) using OpenBabel. Tautomeric states were evaluated and the most energetically favourable forms were retained. These structures were generated through the incorporation of hydrogen atoms, designating torsional bonds, and calculating Gasteiger partial charges. Geometry optimization of the ligands was performed utilizing the Merck molecular force field 1994 (MMFF94) to docking. For molecular dynamics simulations, ligand parameters were created employing the general amber force field 2 (GAFF2). Partial atomic charges were assigned utilizing the AM1-BCC charge model through the antechamber module in AMBER22 software version 22.

#### 4.8.2. Protein Preparation

Prior assessment of binding interaction of chosen metabolites against high-resolution (resolution ≤ 2.5 Å) human crystal structures of BRAF (PDB ID: 4MNF) [64], MAOB (PDB ID: 7P4F) [65], PARP1 (PDB ID: 6I8M) [66], BACE1 (PDB ID: 4XXS) [67], and GSK3β (PDB ID: 5F94) [68], sourced from the Protein Data Bank, were prepared. The structures chosen were based on the following criteria: experimental resolution (≤2.5 Å), human origin, and the completeness of the catalytic domain. Preparation included the removal of crystallographic water molecules beyond 5 Å from active sites, elimination of co-crystallized inhibitor and non-essential heteroatoms, assignment of protonation states at pH 7.4, and addition of missing hydrogen atoms. Protein structures were parameterized using the ff19SB force field. Energy minimization was achieved prior to docking to relieve steric clashes.

#### 4.8.3. Molecular Docking Simulation

Molecular docking was conducted through the utilization of AutoDock Vina (v1.2.3) [69]. Grid boxes were centred on predicted catalytic pockets recognized via P2Rank, with dimensions extending 10 Å beyond active-site residues to confirm conformational flexibility. Binding free energies were calculated using molecular mechanics generalized born surface area (MM-GBSA) over 100 evenly spaced snapshots extracted from the equilibrated phase (100–200 ns). This was done to avoid early structural relaxation and ensure stable conformational sampling. A summation of van der Waals, electrostatic, polar solvation, and nonpolar solvation energies was utilized to determine the total binding free energy (ΔG_bind). The docking pose exhibiting the best estimated binding free energy (ΔG_bind) was chosen for subsequent analysis for every protein–ligand system.

#### 4.8.4. Molecular Dynamics Simulation

AMBER22 software version 22 was utilized for all-atom molecular dynamics simulations. To enable graphics processing unit (GPU) acceleration, pmemd.cuda in AMBER22 software version 22 was utilized. The protein–ligand complexes were solvated in an octahedral TIP3P water box with a minimum distance of 10 Å between the boundary and solute and counterions were utilized for the neutralization process. Energy-optimized systems were warmed from 0 K to 310 K under NVT settings, equilibrated at 310 K and 1 atm, and maintained at NPT conditions, and underwent 200 ns of production simulations using a 2 fs time step. The particle mesh Ewald technique was used to handle long-range electrostatics, and trajectories were recorded every 10 ps.

Root-mean-square deviation (RMSD) analysis was performed to evaluate the structural stability of the protein–ligand complexes throughout the simulation trajectories. Protein RMSD was calculated using backbone Cα atoms following least-squares fitting to the initial structure. Structural alignment was performed using backbone atoms (N, Cα, and C), providing a consistent reference frame for trajectory comparison. This approach enables reliable assessment of global protein stability while minimizing contributions from highly flexible side chain atoms, which were excluded from RMSD calculations due to their intrinsic mobility. Ligand RMSD was calculated using all heavy atoms after alignment to the protein backbone (Cα-based alignment), allowing for evaluation of ligand positional stability relative to the equilibrated protein structure.

Trajectory analysis was conducted using the CPPTRAJ module in AMBER22 software version 22. RMSD stability, hydrogen bond occupancy, and residue interaction mapping were evaluated. RMSD profiles of protein backbone (Cα atoms) and ligand (heavy atoms) were analyzed to determine structural convergence. Stable RMSD trends were interpreted as indicative of sustained ligand binding and conformational stability of the protein–ligand complexes.

### 4.9. In Vitro Evaluation

#### Reduced Neuroblastoma Cell Viability Assay (CCK-8)

The evaluation of the chosen compounds for anti-neuroblastoma efficacy was conducted through a cell viability assay known as the Cell Counting Kit-8 (CCK-8, Dojindo, Japan, Cat. No. CK04) following the manufacturer’s instructions. SH-SY5Y human neuroblastoma cells sourced from Cellonex, Roodepoort, South Africa, were collected from a T-25 flask that was roughly 80% confluent using standardized trypsinization methods. Cells were seeded at a density of 2 × 10^4^ cells per well prior to treatment into a 96-well plate and allowed to incubate for 24 h at 37 °C in a humidified environment with 5% CO_2_ to facilitate cell adhesion. The CCK-8 assay measures relative metabolic activity corresponding to viable cell populations rather than absolute cell numbers. Cells were cultured in DMEM supplemented with 10% (*v*/*v*) heat-inactivated fetal bovine serum. Post incubation, culture medium (DMEM with 10% (*v*/*v*) heat-inactivated fetal bovine serum) was gently aspirated, and cells were carefully rinsed once with pre-warmed PBS to remove residual serum components and phenol red that could interfere with compound exposure and absorbance measurements. The rinsing was performed gently without direct pipette contact with the monolayer to minimize mechanical cell loss. The cells were treated with the tested compounds at a volume of 100 µL: Lactol, Amino(1H-indol-2-yl)acetic acid, Quercetin 3-(6″-malonyl-glucoside), and Doxorubicin (positive control) at concentrations of 0.03 µM, 1 µM, 3 µM, 10 µM, 30 µM, and 100 µM. All compounds were sourced from Sigma-Aldrich, Merck (St. Louis, Missouri, USA) with purity of ≥95% (HPLC grade).

The compounds were initially dissolved in DMSO to create stock solutions of 10 mM, which were subsequently diluted in medium, maintaining a final DMSO concentration of no more than 0.1% (*v*/*v*). A vehicle control containing 0.1% (*v*/*v*) DMSO was included to account for solvent-related effects. The negative control comprised untreated cell culture medium, while wells containing medium and CCK-8 reagent without cells served as blanks. Compounds were administered in complete DMEM supplemented with 10% (*v*/*v*) heat-inactivated fetal bovine serum during the 24 h exposure period to maintain standard culture conditions and cellular stability. The 24 h exposure period was selected to assess early cytotoxic and antiproliferative responses of SH-SY5Y neuroblastoma cells to the tested compounds while minimizing secondary adaptive responses that might arise during longer incubation periods [70]. Following a period of 24 h post-compound-exposure, 10 µL of CCK-8 reagent was placed in each well, and the plate was subjected to an incubation period of an additional 2 h. The absorbance was recorded with a microplate reader (Infinite M200, Tecan, Switzerland) at 450 nm. All CCK-8 experiments were performed using two independent biological replicates, with each treatment condition assessed in technical triplicate. Cell viability (%) was calculated using the following equation

$$\mathrm{Cell}\;\mathrm{viability}\;(\%)=\left(\frac{A_{\mathrm{sample}}-A_{\mathrm{blank}}}{A_{\mathrm{control}}-A_{\mathrm{blank}}}\right)\times 100$$

where $A_{\mathrm{sample}}$ is the absorbance of treated cells, $A_{\mathrm{control}}$ is the absorbance of untreated control cells, and $A_{\mathrm{blank}}$ is the absorbance of the blank wells.

In addition, GraphPad Prism 10 (GraphPad Software, La Jolla, CA, USA) was used to analyze the cell viability data, and the results were represented by dose–response curves, which were created through nonlinear regression analysis (inhibitor vs. response), allowing for automatic determination of IC_50_ values. For IC_50_ determination, technical replicates were averaged within each biological replicate prior to nonlinear regression analysis.

## 5. Conclusions

Transcriptomic profiling of doxorubicin-treated SH-SY5Y neuroblastoma cells revealed stress-adaptive signalling pathways including *BRAF*, *GSK3β*, *BACE1*, *MAOB*, and *PARP1*. Structure-based molecular docking and molecular dynamics simulations suggest that selected plant-derived metabolites may interact with these targets, and in vitro CCK-8 assays proved concentration-dependent reductions in cell viability. Although these findings provide preliminary understanding of potential interactions between plant metabolites and stress-adaptive signalling pathways in neuroblastoma cells, additional mechanistic studies and validation in broader experimental systems will be essential to determine their therapeutic relevance.

## Figures and Tables

**Table 1:** Top 20 gene targets driving KEGG pathway enrichment in doxorubicin-treated SH-SY5Y cells.

Rank	Gene ID	Pathways	Adjusted *p*-Value (FDR)	log_2_FC	FPKM	Pathway Relevance	Score
1	*APP*	Alzheimer disease	1.20 × 10^−8^	+1.396	50.00	1.0	5.862
2	*FGFR3*	Cancer pathway	4.56 × 10^−9^	−2.146	3.99	0.5	4.966
3	*BRAF*	Cancer pathway	4.56 × 10^−9^	−1.528	3.99	0.5	4.966
4	*AKT1*	Cancer pathway	1.89 × 10^−9^	−1.005	1.99	0.5	4.936
5	*MTOR*	Cancer pathway	1.89 × 10^−9^	−1.180	1.99	0.5	4.936
6	*PSEN1*	Alzheimer disease	3.50 × 10^−7^	+1.009	20.50	1.0	4.756
7	*NTRK1*	Neurodegeneration	1.23 × 10^−8^	−1.375	2.15	1.0	4.652
8	*MEK1*	Cancer pathway	1.23 × 10^−8^	−0.847	2.15	0.5	4.552
9	*VEGFA*	Angiogenesis	2.50 × 10^−7^	−0.663	4.20	0.5	4.115
10	*MAPT*	Neurodegeneration	4.20 × 10^−6^	−0.620	15.00	1.0	4.088
11	*MAOB*	Neurodegeneration	7.89 × 10^−7^	−1.240	5.07	1.0	4.032
12	*AGRN*	Neurodegeneration	7.89 × 10^−7^	−0.620	5.07	1.0	4.032
13	*EGFR*	Cancer pathway	6.78 × 10^−8^	−0.797	0.57	0.5	3.880
14	*PARP1*	DNA repair	6.78 × 10^−8^	+1.003	0.57	0.5	3.880
15	*CDK5*	Neurodegeneration	9.00 × 10^−7^	+1.384	1.20	1.0	3.564
16	*BACE1*	Alzheimer disease	7.80 × 10^−6^	−2.117	5.50	1.0	3.564
17	*SNCA*	Neurodegeneration	2.10 × 10^−5^	+0.824	8.00	1.0	3.490
18	*GSK3β*	Alzheimer disease	4.07 × 10^−7^	−2.043	0.16	1.0	3.459
19	*HES4*	Neurodegeneration	4.07 × 10^−7^	−0.889	0.16	1.0	3.459
20	*SOD2*	Oxidative stress	5.00 × 10^−6^	−0.877	3.00	0.5	3.350

Key: Highlighted genes were subsequently prioritized as molecular docking targets.

**Table 2:** Metabolites identified from metabolomic profiling and evaluated through molecular docking against the prioritized targets.

Compound Name	Compound ID	Formula	*m*/*z*	RT [min]	Docking Score	Binding Free Energy ΔGbind (kcal/mol)
MAO B						
Lactol	HMDB0303945	C_20_H_24_O_6_	378.19013	3.939	−9.698	−33.86
4′,5,7-Trihydroxy-6-prenylflavanone	HMDB0037247	C_20_H_20_O_5_	358.16398	3.227	−8.848	−48.29
3′-Deoxythymidine	HMDB0246094	C_10_H_14_N_2_O_4_	227.1062	5.839	−8.032	−35.119
Cibaric acid	HMDB0038580	C_18_H_28_O_5_	366.22678	4.971	−7.905	−54.944
3′-Amino-3′-deoxythimidine	HMDB0060750	C_10_H_15_N_3_O_4_	242.11701	4.038	−7.744	−39.218
2′,3′-Dideoxyuridine	HMDB0245547	C_9_H_12_N_2_O_4_	213.09057	4.976	−7.632	−41.707
Caryatin glucoside	HMDB0037352	C_24_H_26_O_12_	529.13309	4.025	−7.566	−30.277
Biocytin	HMDB0003134	C_16_H_28_N_4_O_4_S	414.21116	4.469	−7.476	−59.884
Amino(1H-indol-2-yl)acetic acid	CSID32887546	C_10_H_10_N_2_O_2_	191.08118	3.17	−7.463	−41.74
Thymidine	HMDB0000273	C_10_H_14_N_2_O_5_	243.10105	5.406	−7.326	−31.752
BRAF						
Lactol	HMDB0303945	C_20_H_24_O_6_	378.19013	3.939	−7.931	−64.179
6″-O-Acetylglycitin	HMDB0039489	C_24_H_24_O_11_	489.13817	5.524	−7.802	−77.906
Aloesol 7-glucoside	HMDB0040565	C_19_H_24_O_9_	438.17503	4.058	−7.778	−57.564
4′,5,7-Trihydroxy-6-prenylflavanone	HMDB0037247	C_20_H_20_O_5_	358.16398	3.227	−7.454	−53.497
5-Methyldeoxycytidine	HMDB0002224	C_10_H_15_N_3_O_4_	283.13964	2.036	−7.316	−46.352
6-Ketoestriol	HMDB0000530	C_18_H_22_O_4_	344.18475	6.526	−7.183	−45.055
Sergliflozin A	HMDB0258246	C_20_H_24_O_7_	418.18493	3.22	−7.058	−53.923
Lactucin	HMDB0035814	C_15_H_16_O_5_	318.13286	3.633	−6.893	−36.205
2′,3′-Dideoxyadenosine	HMDB0245544	C_10_H_13_N_5_O_2_	277.14047	6.718	−6.769	−51.332
Nicotine glucuronide	HMDB0001272	C_16_H_22_N_2_O_6_	356.18075	1.84	−6.601	−50.498
BACE1						
2″-O-Acetylrutin	HMDB0039929	C_29_H_32_O_17_	691.12656	8.193	−6.13	−51.487
Fluocinolone	HMDB0252347	C_21_H_26_F_2_O_6_	454.20623	2.966	−5.883	−54.185
Amino(1H-indol-2-yl)acetic acid	CSID32887546	C_10_H_10_N_2_O_2_	191.08118	3.17	−5.715	−40.305
Quercetin 3-(6″-malonyl-glucoside)	HMDB0037368	C_24_H_22_O_15_	551.10198	4.937	−5.694	−72.447
Aloesol 7-glucoside	HMDB0040565	C_19_H_24_O_9_	438.17503	4.058	−5.693	−55.974
3′-Deoxythymidine	HMDB0246094	C_10_H_14_N_2_O_4_	227.1062	5.839	−5.623	−37.631
Aflatoxin G2	HMDB0030475	C_17_H_14_O_7_	331.08041	6.708	−5.621	−45.966
Sergliflozin A	HMDB0258246	C_20_H_24_O_7_	418.18493	3.22	−5.554	−59.878
25-Hydroxyvitamin D3-26,23-lactone	HMDB0060126	C_27_H_40_O_4_	451.28331	11.375	−5.381	−50.272
Lactucin	HMDB0035814	C_15_H_16_O_5_	318.13286	3.633	−5.277	−45.832
GSK3β						
Amino(1H-indol-2-yl)acetic acid	CSID32887546	C_10_H_10_N_2_O_2_	191.08118	3.17	−7.872	−49.268
2′,3′-Dideoxyadenosine	HMDB0245544	C_10_H_13_N_5_O_2_	277.14047	6.718	−7.168	−46.263
7-Methylinosine	HMDB0003950	C_11_H_15_N_4_O_5_	325.139	1.031	−6.693	−51.859
6-Ketoestriol	HMDB0000530	C_18_H_22_O_4_	344.18475	6.526	−6.65	−55.158
Protocatechuic acid 4-glucoside	HMDB0303826	C_13_H_16_O_9_	339.06788	2.983	−6.625	−59.802
Nicotine glucuronide	HMDB0001272	C_16_H_22_N_2_O_6_	356.18075	1.84	−6.571	−58.084
Aloesol 7-glucoside	HMDB0040565	C_19_H_24_O_9_	438.17503	4.058	−6.565	−68.261
Sergliflozin A	HMDB0258246	C_20_H_24_O_7_	418.18493	3.22	−6.108	−38.478
Lactol	HMDB0303945	C_20_H_24_O_6_	378.19013	3.939	−6.014	−32.732
5-Methyldeoxycytidine	HMDB0002224	C_10_H_15_N_3_O_4_	283.13964	2.036	−5.923	−41.794
PARP1						
3′-Deoxythymidine	HMDB0246094	C_10_H_14_N_2_O_4_	227.1062	5.839	−7.791	−45.312
Aloesol 7-glucoside	HMDB0040565	C_19_H_24_O_9_	438.17503	4.058	−7.701	−72.594
Quercetin 3-(6″-malonyl-glucoside)	HMDB0037368	C_24_H_22_O_15_	551.10198	4.937	−7.454	−95.369
7-Methylinosine	HMDB0003950	C_11_H_15_N_4_O_5_	325.139	1.031	−7.255	−69.042
Quercetin 3-O-(6″-acetyl-glucoside)	HMDB0029271	C_23_H_22_O_13_	507.11242	3.718	−7.172	−85.366
2″-O-Acetylrutin	HMDB0039929	C_29_H_32_O_17_	691.12656	8.193	−7.07	−69.72
Pyroglutamylvaline	HMDB0094651	C_10_H_16_N_2_O_4_	229.12191	5.782	−7.051	−51.081
Zeranol	HMDB0032702	C_18_H_26_O_5_	364.2109	4.259	−6.869	−55.965
Kasugamycin	CSID16736502	C_14_H_25_N_3_O_9_	402.14996	1.915	−6.738	−73.803
Nicotine glucuronide	HMDB0001272	C_16_H_22_N_2_O_6_	356.18075	1.84	−6.665	−57.541

Key: Highlighted top 3 prioritized metabolites which were processed further for molecular dynamics analysis. The calculated binding free energy values (ΔGbind) correspond to computational binding affinity estimates derived from MM-GBSA calculations performed on equilibrated molecular dynamics trajectories.

## Data Availability

The original contributions presented in the study are included in the article; further inquiries can be directed to the corresponding author.

## Supplementary Materials

The following supporting information can be downloaded at https://www.mdpi.com/article/10.3390/ijms27093739/s1.

## Abbreviations

The following abbreviations are used in this manuscript:

AGRN	Agrin
AKT1	AKT serine/threonine kinase 1
APP	Amyloid beta precursor protein
BACE1	Beta-site amyloid precursor protein cleaving enzyme 1
BRAF	B-Raf proto-oncogene, serine/threonine kinase
CCK-8	Cell Counting Kit-8
CDK5	Cyclin-dependent kinase 5
cDNA	Complementary Deoxyribonucleic acid
CO_2_	Carbon dioxide
DMEM	Dulbecco’s Modified Eagle Medium
DMSO	Dimethyl sulfoxide
DNA	Deoxyribonucleic acid
EDTA	Ethylenediaminetetraacetic acid
EGFR	Epidermal growth factor receptor
ESI^+^	Positive electrospray ionization mode
FC	Fold Change
FDR	False discovery rate
FGFR3	Fibroblast growth factor receptor 3
FPKM	Fragments per kilobase of transcript per million mapped reads
GAFF2	General Amber Force Field 2
GSK3β	Glycogen synthase kinase 3 beta
HES4	Hes family bHLH transcription factor 4
HMDB	Human Metabolome Database
KEGG	Kyoto Encyclopedia of Genes and Genomes
MAOB	Monoamine oxidase B
MAPT	Microtubule-associated protein tau
MEK1	Mitogen-activated protein kinase 1
MM-GBSA	Molecular mechanics–generalized Born surface area
MTOR	Mechanistic target of rapamycin kinase
*m*/*z*	mass-to-charge ratio
NTRK1	Neurotrophic receptor tyrosine kinase 1
NPT	Constant number of particles, pressure, and temperature ensemble
NVT	Constant number of particles, volume, and temperature ensemble
P1–Pn	Predicted ligand-binding pockets
PARP1	Poly(ADP-ribose) polymerase 1
PBS	Phosphate-buffered saline
PDB	Protein Data Bank
PCR	Polymerase chain reaction
PPI	Protein–protein interaction
PSEN1	Presenilin 1
RINe	RNA integrity number equivalent
RMSD	Root mean square deviation
RNA	Ribonucleic acid
RNA-seq	Ribonucleic acid sequencing
RT	Retention Time
SOD2	Superoxide dismutase 2 (mitochondrial)
SNCA	Synuclein alpha
TIP3P	Transferable Intermolecular Potential with 3 Points
TSV	Tab-separated values
UPLC-MS/MS	Ultra-performance liquid chromatography–tandem mass spectrometry
VEGFA	Vascular endothelial growth factor A
Wnt	Wingless-related integration site
