# Supplementary material for: Network-Guided Identification of Plant-Derived Modulators of Stress-Adaptive Signalling in Neuroblastoma
Source: Int J Mol Sci. 2026 Apr 23;27(9):3739. doi: 10.3390/ijms27093739 (PMC13163309; doi:10.3390/ijms27093739)
Supplement: Supplementary file 1 [file ijms-27-03739-s001.zip › Supplementary material Figure S1.pdf]

Supplementary material

MAO B

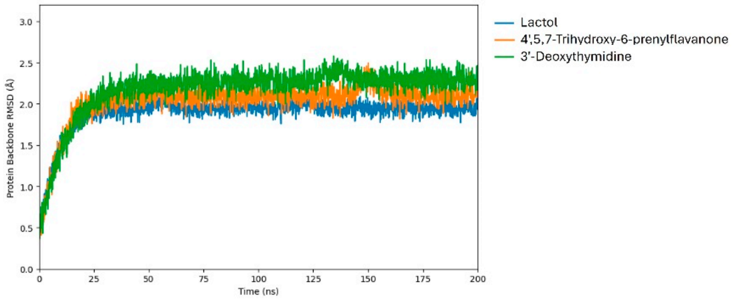

(a)

BRAF

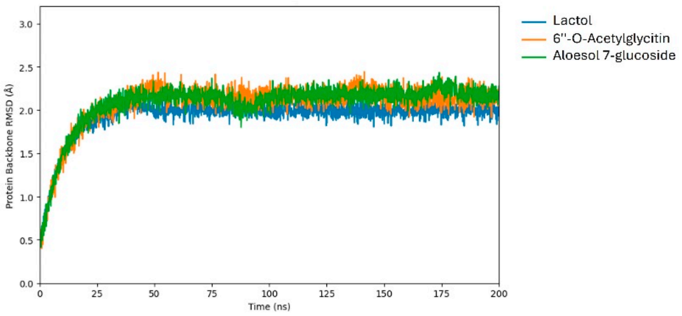

(b)

BACE1

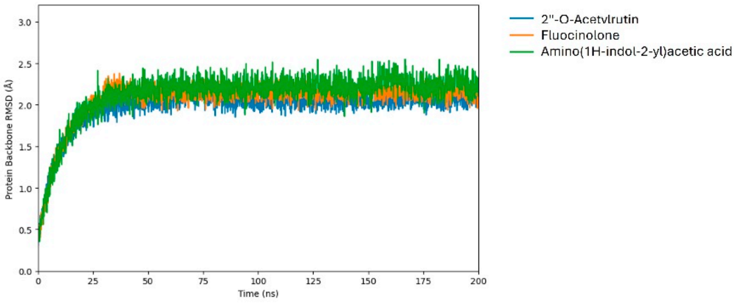

(c)

GSK-3β

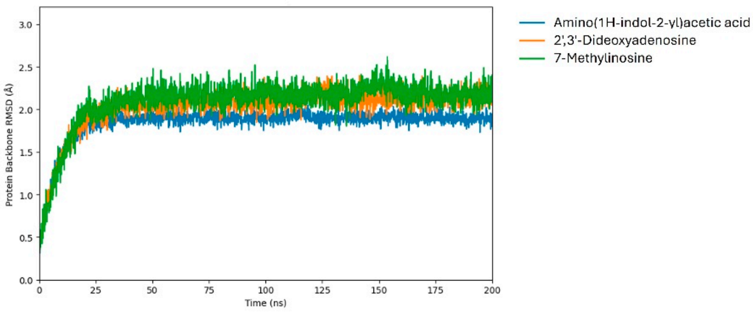

PARP1

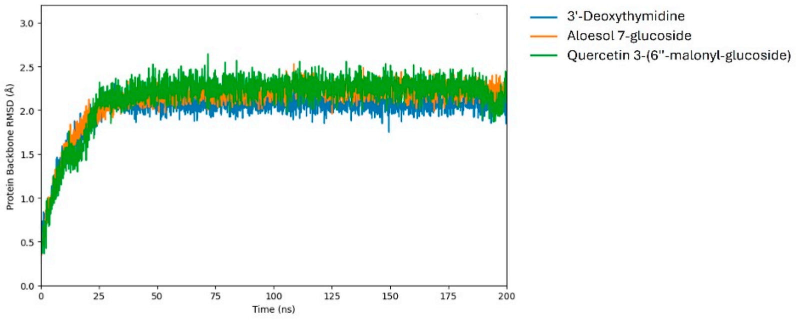

(e)

Figure S1. Root-mean-square deviation (RMSD) plots showing the structural stability of protein-ligand complexes during 200 ns molecular dynamics simulations. Protein RMSD was calculated using backbone Cα atoms following alignment based on backbone atoms (N, Cα, C), while ligand RMSD was calculated using all heavy atoms after alignment to the protein backbone.
